# Sodium-Glucose Cotransporter-2 Inhibitors as Enablers of Chronic Glucocorticoid Therapy

**DOI:** 10.1016/j.jacadv.2023.100343

**Published:** 2023-05-24

**Authors:** Rami Kahwash, Javed Butler

**Affiliations:** a Ohio State University Wexner Medical Center, Columbus, Ohio, USA; b Baylor Scott and White Research Institute, Dallas, Texas, USA; c University of Mississippi, Jackson, Mississippi, USA

**Keywords:** cotransporter, enablers, glucocorticoid, SGLT2-I, sodium-glucose, steroid

Many lifesaving and disease-modifying therapies are associated with significant side effects that limit their use. These can range from difficulty to tolerate therapy (eg, nausea and vomiting with chemotherapy), development of another comorbidity (eg, hypothyroidism with amiodarone), to development of life-threatening side effects (eg, severe hyperkalemia with mineralocorticoid antagonists or gastrointestinal bleeding with dual antiplatelet use). Several drugs and devices may physiologically counter these side effects and help enable treatment with disease-modifying agents. These therapies are sometime referred to as *enablers* and describe either a device-based intervention or a class of drug that allows safe administration of other vital therapeutic interventions. For example, antiemetics help enable chemotherapy use,^1^ proton pump inhibitors enable dual antiplatelet inhibition,^2^ and novel potassium binders support mineralocorticoid steroid therapy.^3^ Cardiac resynchronization therapy and transcatheter edge-to-edge mitral value repair are associated with improved tolerability of heart failure drug therapy.

Glucocorticoids are widely used for their broad-spectrum anti-inflammatory and immunomodulator actions mediated by direct nongenomic and genomic mechanisms involving inhibition or activation of transcription of key regulatory proteins.^4^ Glucocorticoids gained recognition as first-line treatment options of various medical conditions such as allergic reactions, asthma, chronic obstructive lung disease, autoimmune and systemic inflammatory disorders, hematological malignancies, and in post organ transplant recipients. Glucocorticoids are one of the most frequently prescribed drugs worldwide and their use continue to increase. A cross-sectional study from France on a nationwide representative sample showed that 14.7% of adults received at least one prescription of glucocorticoids in 2007; this rate increased to 17.1% in 2014.^5^

Long-term administration of glucocorticoids is often challenged by poor tolerance and adverse effects, including new onset diabetes, worsening of prevalent diabetes, dyslipidemia, weight gain, new or worsening hypertension, peripheral edema, fluid retentions and worsening heart failure, among others.^6^^,^^7^ Glucocorticoids markedly impair quality of life through multiple mechanisms including poor muscle glucose uptake, fatigue, obesity-mediated physical debility as well as direct central nervous system side effects that alter mood and neuropsychiatric health.^6^ Glucocorticoids induced mood and behavioral disorders are seen in up to 1 in 4 patients.^6^^,^^7^ Glucocorticoids predispose to infection, osteoporosis, and fractures with long-term use. Many indications for glucocorticoids therapy are long-term or lifelong, and their side effects often lead to underdosing or premature interruption prior to achieving therapeutic goals. Currently there are no alternatives to glucocorticoids or known therapies to safely enable their use.

Sodium-glucose cotransporter-2 (SGLT2) inhibitors are a novel class of drugs with well-established cardiovascular, renal, and metabolic benefits. SGLT2 inhibitors mechanisms of action are complex and yet to be fully understood, however, some of the well-established mechanisms are well suited to directly combat many of glucocorticoids deleterious biological and emotional side effects. SGLT2 inhibitors enhance urinary sodium and glucose excretion, improve glycemic control through noninsulin-dependent mechanisms, and favorably alter the metabolic milieu leading to weight loss and improved vascular function, culminating in improved cardiovascular outcomes.^8^ While SGLT2 inhibitors’ clinical indications are still unfolding, their known multiple systemic effect allows them to be an ideal glucocorticoids enabler. SGLT2 inhibitors may ameliorate glucocorticoids side effects by direct and indirect mechanisms through normalizing of sodium and glucose homeostasis and restoration of endocrine, metabolic, and vascular function (Figure 1).

**Figure 1:**
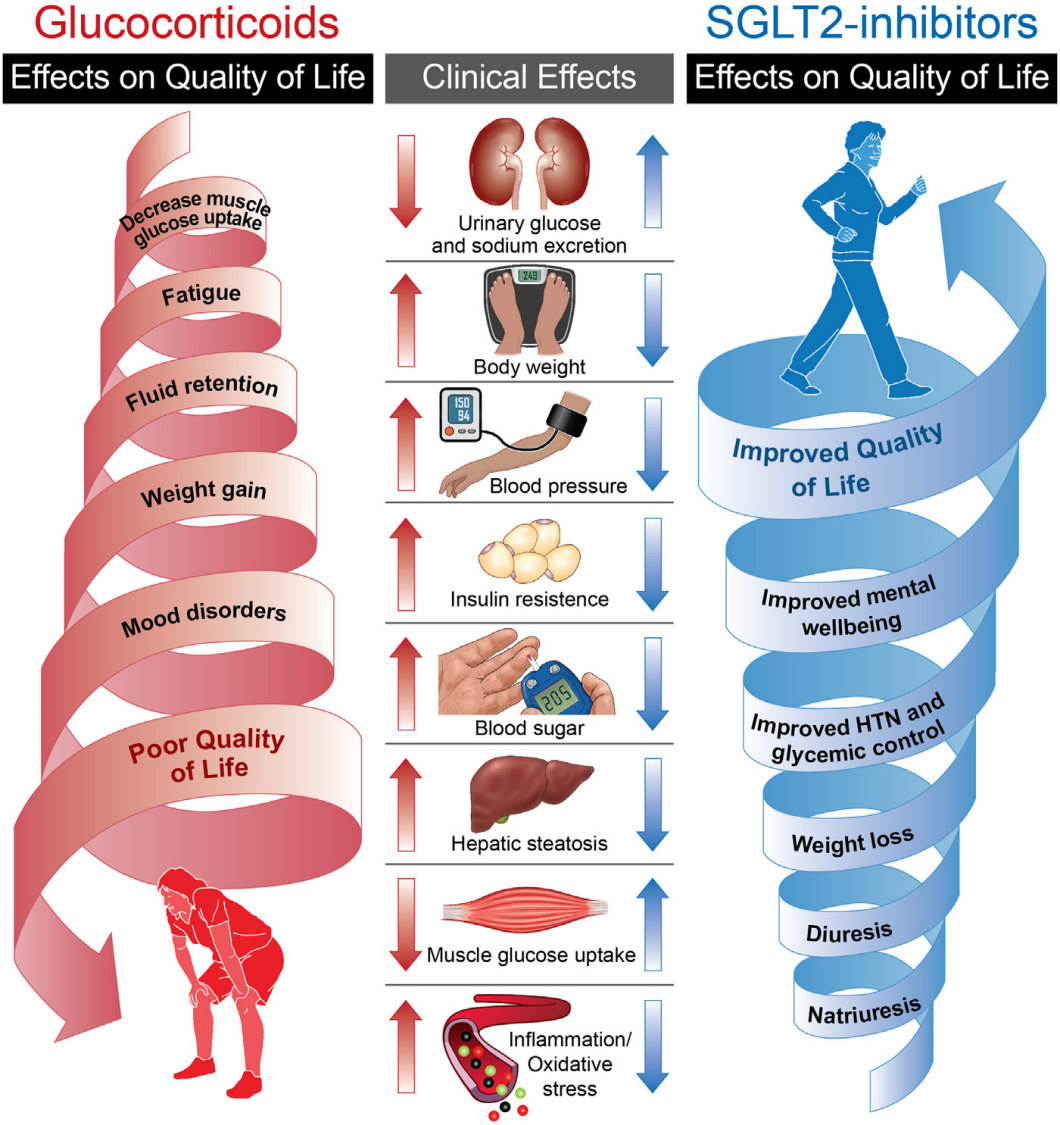
**SGLT2 Inhibitors and Glucocorticoids Opposing Mechanisms** SGLT2 = sodium-glucose cotransporter-2.

Given their safety profile, SGLT2 inhibitors are well positioned to be coadministered to avoid or mitigate glucocorticoids side effects. However, such studies have not been performed to date. Dosing and duration of glucocorticoids treatment remain largely unstandardized and lack strong recommendations by published guidelines. Expected studies should focus on a population with standardized glucocorticoids use with long-term indication. The post solid organ transplant group represents perhaps the ideal study target due to protocol driven glucocorticoids schedules, higher induction dosages, longer treatment exposure, and excellent patient adherence owing to rigorous pretransplant psychosocial screening and need for intensified monitoring. Studies need to be conducted in patients with chronic glucocorticoids use to explore the SGLT2 inhibitors ability to allow longer exposure to glucocorticoids with lesser side effects, fewer treatment interruptions, and unplanned dose reductions, show improvement in patient-centered outcomes through well validated quality of life and health status tool, improved scores on steroid toxicity metrics, and prevent and ameliorate glucocorticoids-related common comorbid conditions such as diabetes, hyperlipidemia, hypertension, and heart failure.

In summary, glucocorticoids are the main first-line therapy for various clinical indications that span nearly all medical subspecialties. Glucocorticoids use is associated with severe side effects in majority of these patients. Current practice has been reactionary and focused on treating glucocorticoids side effects after they occur which is often delayed and associated with clinical and emotional comorbidities, increased health care expenditure, and great negative impact on quality of life. SGLT2 inhibitors have the potential of serving as ideal glucocorticoids enablers that can potentially prevent adverse effects before they occur through biologically opposing mechanisms. Future studies are needed to test SGLT2 inhibitors roles as glucocorticoids enabler.

## Funding support and author disclosures

Dr Kahwash has served as a consultant for Medtronic, Impulse Dynamics, and Cardionomic. Dr Butler has served as a consultant for Abbott, Adrenomed, Amgen, Array, Astra Zeneca, Bayer, Boehringer Ingelheim, Bristol Myers Squib, CVRx, G3 Pharmaceutical, Impulse Dynamics, Innolife, Janssen, LivaNova, Luitpold, Medtronic, Merck, Novartis, NovoNordisk, Relypsa, Roche, V-Wave Limited, and Vifor.
